# Right on target: The next class of efficient, safe, and specific RNAi triggers

**DOI:** 10.1016/j.omtn.2022.03.027

**Published:** 2022-04-12

**Authors:** Jonas Becker, Nico Fischer, Dirk Grimm

**Affiliations:** 1 Department of Infectious Diseases/Virology, Medical Faculty, University of Heidelberg, Center for Integrative Infectious Diseases Research (CIID), BioQuant, 69120 Heidelberg, Germany; 2 Faculty of Biosciences, University of Heidelberg, 69120 Heidelberg, Germany; 3 German Center for Infection Research (DZIF), Partner Site Heidelberg, 69120 Heidelberg, Germany; 4 German Center for Cardiovascular Research (DZHK), Partner Site Heidelberg, 69120 Heidelberg, Germany

**Keywords:** RNA interference, RNAi, shRNA, promoter, miRNA, agshRNA, RISC, lentiviral vectors, safety, specificity

RNA interference (RNAi) is a powerful tool to control the expression of a target gene, yet RNAi triggers, typically small interfering (si) or short hairpin (sh) RNAs, are often prone to inducing adverse side effects that can limit their therapeutic index. By embedding Argonaute 2 (Ago2)-dependent shRNAs (agshRNAs) within a micro (mi)RNA backbone, and by thus enabling tissue-specific expression from an RNA polymerase (pol) II promoter, Alsing and colleagues have now taken a pivotal step at increasing the specificity and safety of therapeutic RNAi. Additionally, an RNA-seq-based comparison permitted the authors to identify side effects of agshRNAs or their conventional shRNA counterparts. Thereby, this study concurrently and compellingly demonstrates the potential to improve RNAi safety and efficiency, as well as the power of assays to monitor sequence-specific or -unspecific side effects. At the same time, this work highlights that the therapeutic window ensuring sufficient and concomitantly safe target gene knockdown may be narrow.

The concept to harness the endogenous RNAi pathway for targeted mRNA silencing by exogenous introduction or expression of artificial RNAi triggers had been established over 2 decades ago. Among the most frequently employed RNAi triggers are shRNAs, which, especially when delivered by lentiviral (LV) or adeno-associated viral vectors, can achieve sustained target knockdowns. Their expression is usually driven by RNA pol III promoters such as U6, which can produce large quantities of RNAi effectors in transduced cells and mediate robust knockdowns. However, in a manner depending on shRNA sequence, abundancy, and other parameters, shRNAs can also induce adverse side effects comprising (1) off-targeting, (2) saturation of the cellular RNAi machinery and competition with endogenous miRNAs, and (3) induction of immune responses, in the worst case culminating in *in vivo* toxicity.^1^ Previous attempts to resolve these concerns and to improve the safety of RNAi applications have greatly benefitted from our understanding of the intracellular processing of natural RNAi triggers, i.e., miRNAs in mammalian cells. Typically, following their expression as primary (pri-)miRNAs, these molecules undergo a two-step maturation. Initially, the Drosha/DGCR8 complex trims the pri-miRNA at the 5′ and 3′ ends of the hairpin stem, yielding a precursor (pre-)miRNA intermediate; the latter is mimicked by shRNAs. Next, Exportin-5 translocates the pre-miRNA (or shRNA) into the cytoplasm, where it is further processed by the RNase III Dicer. The resulting RNA duplex associates with one of four Argonaute proteins (Ago1–4) in the RNA-induced silencing complex RISC, in which the guide RNA strand is maintained for binding to target mRNAs and the passenger strand is degraded. A concern in the case of shRNAs is that the passenger strand is occasionally retained and then induces unwanted inhibition of off-target mRNAs. As shown before, this can be mitigated by utilizing modified guide-only effectors based on miRNA miR451. This miRNA uses a non-canonical, Dicer-independent pathway, in which it is directly processed by Ago2 in the cytoplasm via cleavage of the 3′-hairpin stem at position 10/11, followed by trimming by the poly(A)-specific ribonuclease (PARN). MiR451-like effectors were found to be independent of Ago1, Ago3, or Ago4, which decreases their competition for endogenous miRNAs and their potential for off-targeting, and in turn enhances their safety.^2^ To date, several versions of artificial miR451 mimics have been reported, having in common a shortened stem sequence and a 1–3 nt 3′ overhang. Ago2-sliced agshRNAs, for instance, use a guide sequence that forms a 17- to 18-bp stem plus a 4-nt loop.^3^

In their current study, Alsing and colleagues generated and directly compared *Vegfa*-targeting shRNAs as well as corresponding agshRNAs based on miR451 or miR324. As hoped for, in cultured cells, the agshRNAs showed (1) no detectable passenger-strand activity against an antisense target, (2) decreased competition with endogenous miRNAs, and (3) partially reduced toxicity after LV transduction compared to their shRNA counterparts. Both, competition and toxicity, were more strongly influenced by the specific target sequence than by the structure of the RNAi effector. Interestingly, both side effects were abolished when the expression of agshRNAs was switched from the U6 to an RNA pol II promoter, by embedding agshRNAs in a miRNA backbone (miR-agshRNAs). Alas, this came at the cost of a decrease in knockdown efficiency. To demonstrate the *in vivo* applicability of miR-agshRNAs, Alsing et al. chose the RPE-specific VMD2 promoter they had used for miRNA-based knockdown of *Vegfa* before.^4^^,^^5^ Subretinal injections of the ensuing LV vector in mice elicited significant target knockdown of *Vegfa* in transduced compared to non-transduced cells. However, the overall transduction efficiency was too low to allow detection of an effector-induced knockdown without a prior fluorescence-activated cell sorting-based isolation of transduced cells.

To moreover study the impact on the cellular transcriptome, Alsing et al. transduced ARPE19 cells with LV vectors containing U6 promoter-driven expression cassettes for three corresponding shRNA/agshRNA pairs. Using RNAseq combined with clustering of differentially regulated genes, this enabled the identification of several side effects elicited by the different effectors through gene ontology enrichment and heptamer seed match analyses. While these results further exemplify the benefits of agshRNAs, such as reduced off-target knockdowns mediated by the passenger-strand seed, they also revealed sequence-specific off-target effects that are shared between sh- and agshRNAs with a common guide strand.

**Figure 1:**
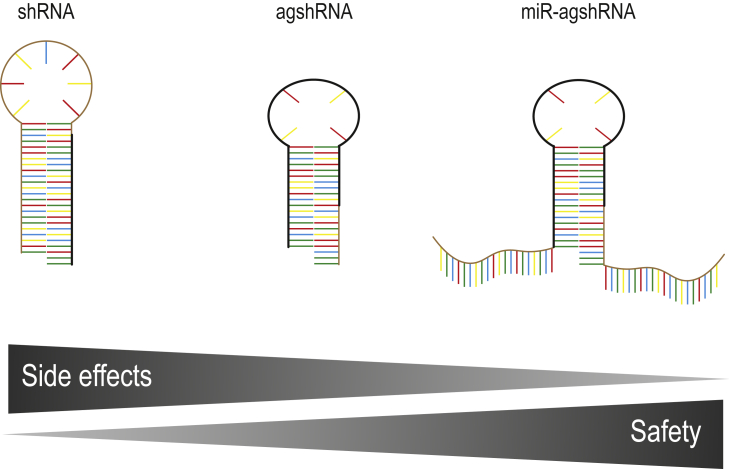
Comparison of the structures and features of the three types of RNAi triggers discussed in this article, i.e., from left to right: conventional shRNA, agshRNA, and the latest miR-agshRNA design. Guide strands are highlighted by thick black lines.

Overall, this work perfectly illustrates the remarkable advances that are constantly made in the RNAi field and that will ultimately help to close the gap in its clinical translation. Coming from (1) classical U6 promoter-driven shRNAs and their propensity for *in vivo* toxicity, followed by (2) agshRNAs with reduced passenger-strand activity and endogenous miRNA competition, the new class of (3) miRNA-embedded agshRNAs driven from tissue-specific RNA pol II promoters reported here promises yet another leap in RNAi specificity and safety (Figure 1). Still, many of the observed side effects were found to depend on the guide strand sequence, so future iterations are well advised to compare multiple different effector sequences to identify candidates combining strong on-target knockdown with minimal side effects and to elucidate the underlying design rules. Notably, the current authors have successfully proved the value of transcriptome analysis for stratification, allowing comprehensive insights into the side effects elicited by a given RNAi effector. In the future, it will be interesting and informative to also include a non-effector control as a reference for inherent transcriptomic changes elicited by RNA pol II- or pol III-driven RNAi systems. Finally, as demonstrated by the *in vivo* application of VMD2 promoter-driven miR-agshRNAs for *Vegfa* knockdown via LV vectors, achieving therapeutic knockdown requires highly efficient vectors as well as strong RNAi effectors and robust expression systems. It has become clear that each of these components necessitates crucial dissection, balancing, and fine-tuning to juxtapose sufficient knockdown efficiency with maximum safety. To this end, it is one of the merits of this study that we now have an expanded toolset available to study, compare, and optimize all these parameters *in vitro* and *in vivo*, which will eventually help to accelerate clinical RNAi translation.
